# Supplementary material for: Regional adaptation defines sensitivity to future ocean acidification
Source: Nat Commun. 2017 Jan 9;8:13994. doi: 10.1038/ncomms13994 (PMC5227702; doi:10.1038/ncomms13994)
Supplement: Supplementary Information — Supplementary Figures, Supplementary Tables and Supplementary References [file ncomms13994-s1.pdf]

Supplementary Figures

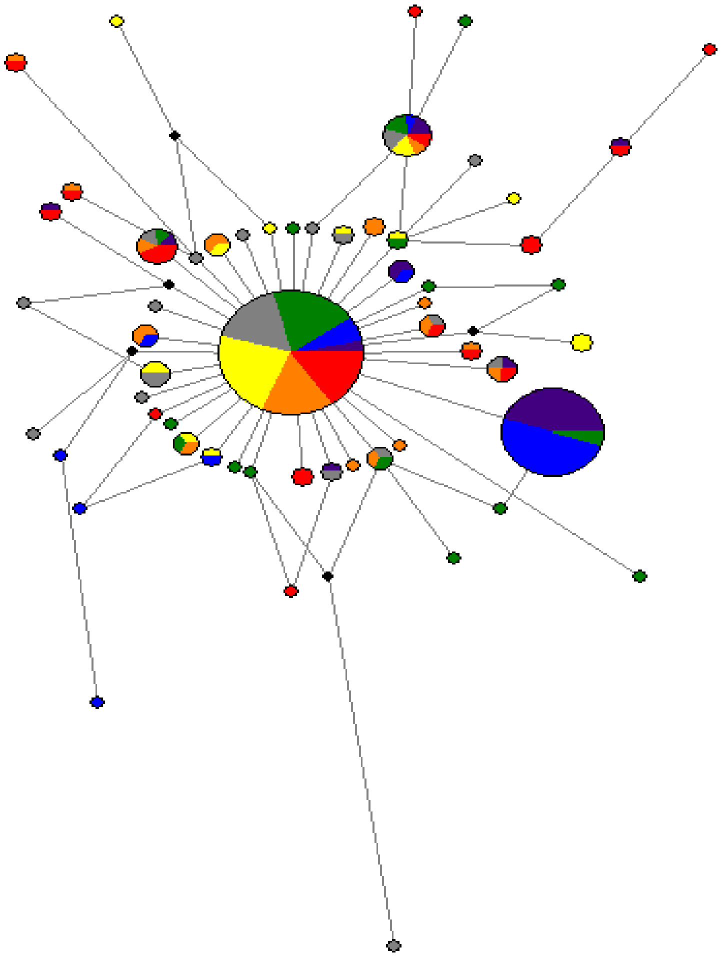

Purple: Tromso
Blue: Trondheim
Green: Millport
Grey: Plymouth
Yellow: Roscoff
Orange: Île de Ré
Red: Vigo

Supplementary Figure 1 | Haplotype network for individuals of *L. littorea*. Haplotype network constructed using the ‘median joining’ method and parsimony showing 57 COI haplotypes (from 238 individuals) of *L. littorea*. Circles represent haplotypes, with the area of each circle representative of the frequency with which it occurred in the whole sample set, and pie charts indicate the distribution of the haplotype among the various populations. Connecting lines show mutational pathways among haplotypes. Black circles show missing intermediaries.

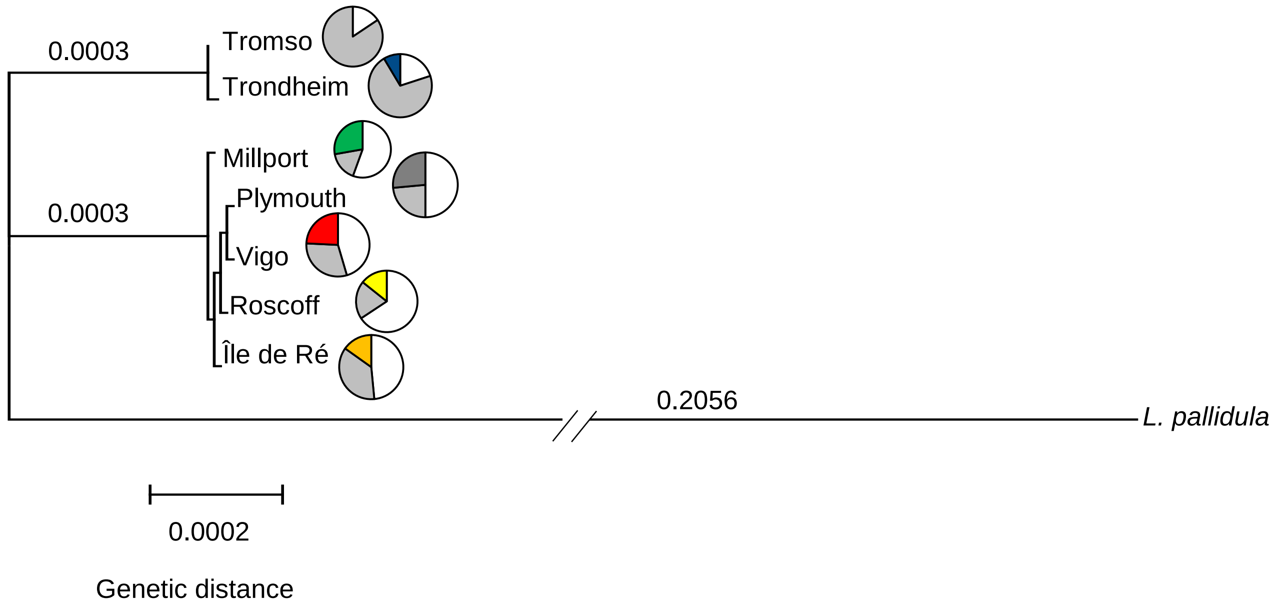

Supplementary Figure 2 | Neighbour joining population tree for *L. littorea.*
Tree based on COI sequences from the different populations of *L. littorea* sampled on a latitudinal gradient in northwest Europe. *Lacuna pallidula* was used as an outgroup. Numbers on branches and lengths of branches are proportional to genetic (D_a) distances. Pie charts for each site represent the proportion of haplotypes that are common to all sites (white), shared between several sites (grey), or private to each site (colours).

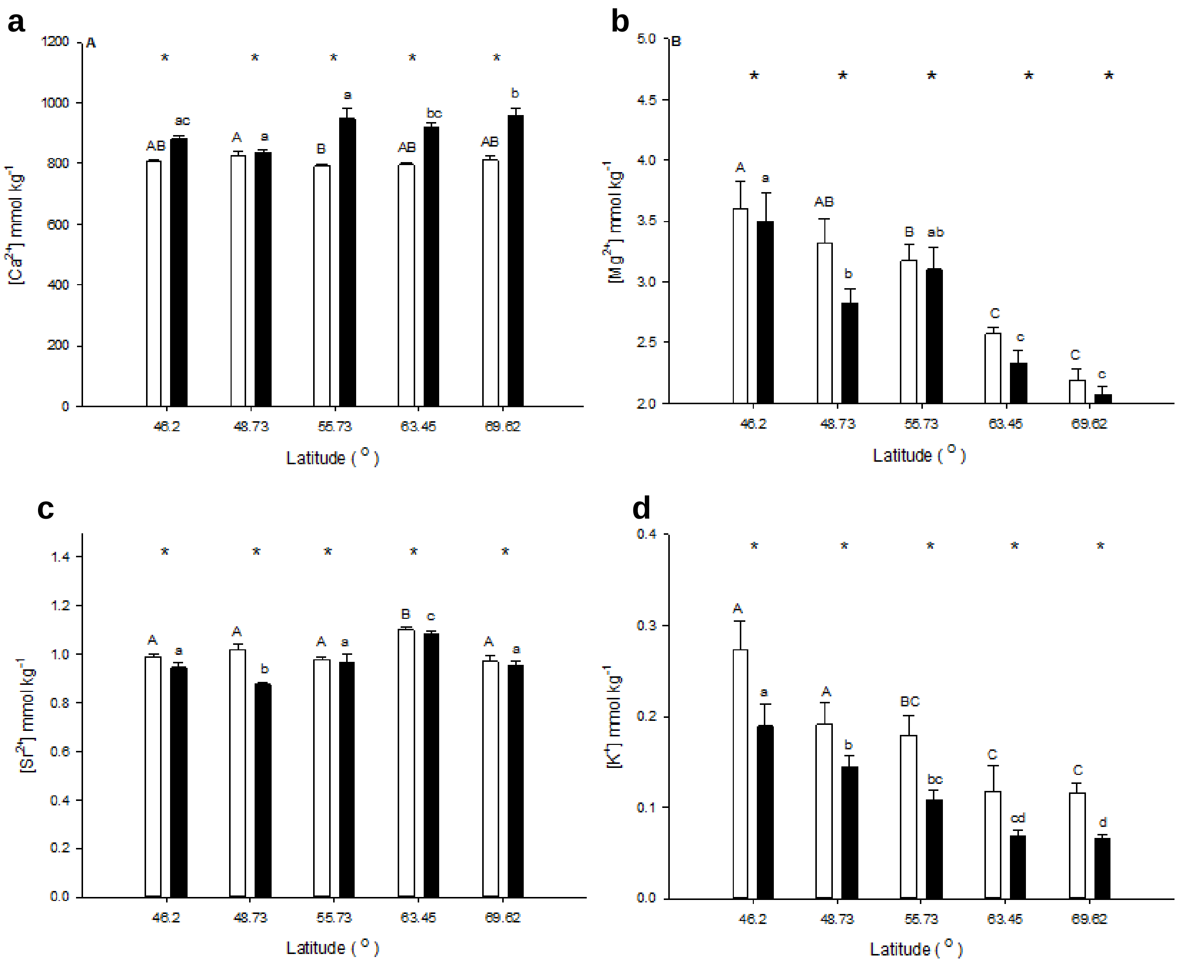

Supplementary Figure 3 | Shell ion concentrations of *L. littorea* populations. Mean (± SE) ion concentrations of empty shells (population means, n=30) (population collection location latitudinal position provided along the x axis) exposed to conditions of current (white) and future P_{CO2} (black). **a,** $[Ca^{2+}]$, **b,** $[Mg^{2+}]$, **c,** $[Sr^{2+}]$, **d,** $[K^{+}]$. * indicates there is a significant difference between the current and future P_{CO2}. Significantly different means ($p \leq 0.05$) within a P_{CO2} treatment are indicated by capital letters for current P_{CO2} conditions *and by* lower case letters for future P_{CO2} conditions (univariate ANOVA and Post Hoc Bonferroni test of one-way ANOVA ($p < 0.05$), according to 95% Confidence Interval test for Estimate Marginal Means (EMM) with Bonferroni).

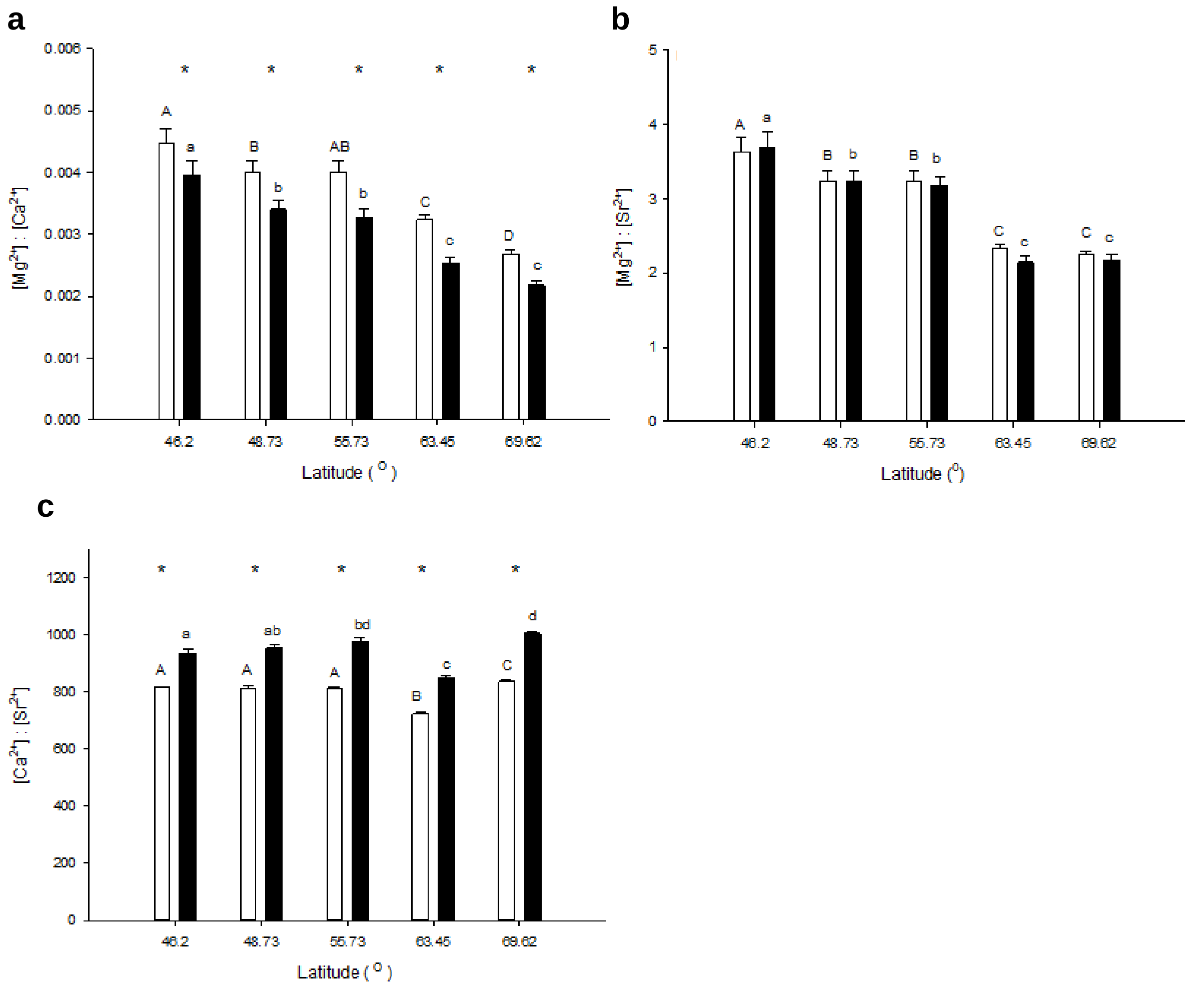

Supplementary Figure 4 | Shell ion concentration ratios of *L. littorea* populations. Mean (± SE) ion concentrations of empty shells (population means, n=30) (population collection location latitudinal position provided along the x axis) exposed to conditions of current (white) and future P_{CO2} (black). **a,** $[Mg^{2+}]$: $[Ca^{2+}]$, **b,** $[Ca^{2+}]$: $[Sr^{2+}]$ and **c,** $[Mg^{2+}]$: $[Sr^{2+}]$. * indicates there is a significant difference between the current and future P_{CO2}. Significantly different means ($p \leq 0.05$) within a P_{CO2} treatment are indicated by capital letters for current P_{CO2} conditions and by lower case letters for future P_{CO2} conditions (univariate ANOVA test and Post Hoc Bonferroni test of one-way ANOVA ($p < 0.05$), according to 95% Confidence Interval test for Estimate Marginal Means (EMM) with Bonferroni correction).

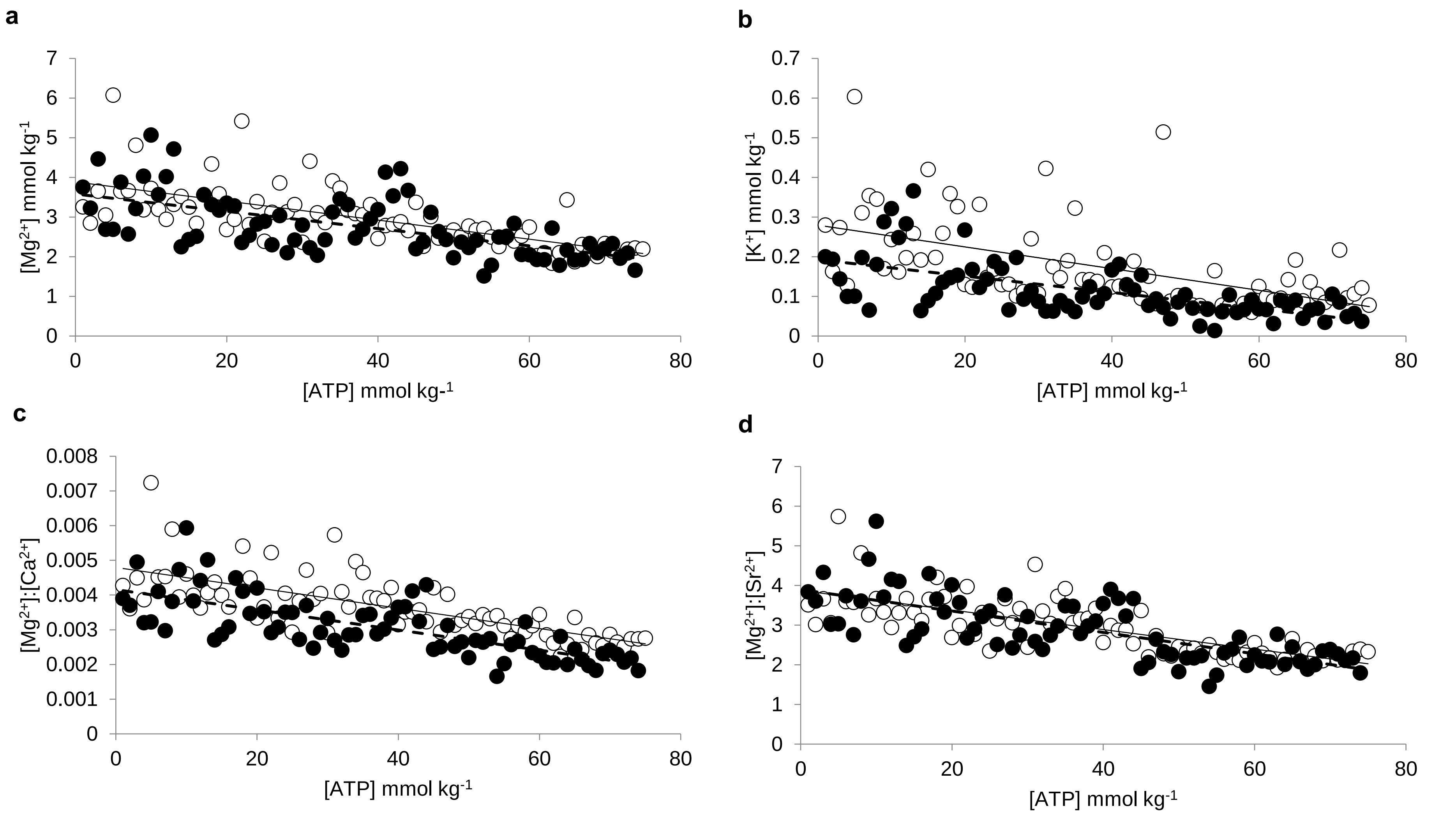

Supplementary Figure 5 | Relationships between snails' [ATP] and shells ion concentrations of *L. littorea* populations.
a, [Mg^{2+}], current $P\text{co}_2$, Pearson's correlation coefficient test $r_{17} = 0.68$, $P = 0.002$, elevated $P\text{co}_2$, $r_{20} = 0.61$, $P = 0.002$.
b, [K^{+}], current $P\text{co}_2$, Pearson's correlation coefficient test $r_{17} = 0.55$, $P = 0.016$, elevated $P\text{co}_2$, $r_{20} = 0.63$, $P = 0.002$.
c, [Mg^{2+}] : [Ca^{2+}], current $P\text{co}_2$, Pearson's correlation coefficient test $r_{17} = 0.71$, $P < 0.001$, elevated $P\text{co}_2$, $r_{20} = 0.73$, $P < 0.001$.
and **d,** [Mg^{2+}] : [Sr^{2+}], current $P\text{co}_2$, Pearson's correlation coefficient test $r_{17} = 0.74$, $P < 0.001$, elevated $P\text{co}_2$, $r_{20} = 0.72$, $P < 0.00$.
(population means, n=100). White circles-dotted line = current $P\text{co}_2$, black circles-full line = elevated $P\text{co}_2$.

Supplementary Table 1 | Sample size, molecular diversity, and population demographic statistics for the common periwinkle *L. littorea* for populations from each sample site for the COI gene. Trm-Tromso, Trd-Trondheim, Mil-Millport, Ply-Plymouth, Ros-Roscoff, IdR-Île de Ré, Vig-Vigo.

	All samples	Trm	Trd	Mil	Ply	Ros	IdR	Vig
Sample size (N)	238	32	35	36	34	35	33	33
Number of haplotypes (N_{hap})	57	9	9	17	18	12	16	16
Haplotype diversity (H_e) (SD)	0.814 ± 0.052	0.607 ± 0.098	0.587 ± 0.090	0.754 ± 0.079	0.809 ± 0.070	0.642 ± 0.093	0.796 ± 0.073	0.818 ± 0.067
Number of polymorphic sites (N_{ps})	54	11	11	17	22	14	18	19
Average number of differences (SD)	1.601 ± 0.954	1.339 ± 0.853	1.21 ± 0.792	1.449 ± 0.902	1.670 ± 1.005	1.220 ± 0.795	1.318 ± 0.843	1.939 ± 1.129
Nucleotide diversity (SD)	0.030 ± 0.020	0.025 ± 0.018	0.022 ± 0.016	0.027 ± 0.019	0.031 ± 0.021	0.023 ± 0.016	0.024 ± 0.017	0.036 ± 0.023

Supplementary Table 2 | Pairwise Φ_{ST} (genetic variance) among sites for the COI gene in the common periwinkle *L. littorea*. Trm-Tromso, Trd-Trondheim, Mil-Millport, Ply- Plymouth, Ros-Roscoff, IdR-Île de Ré, Vig-Vigo.

	Trm	Trd	Mil	Ply	Ros	IdR	Vig
Trm							
Trd	0.000						
Mil	0.168*	0.185*					
Ply	0.199*	0.216*	0.003				
Ros	0.230*	0.243*	0.000	0.000			
IdR	0.224*	0.236*	0.001	0.009	0.006		
Vig	0.189*	0.216*	0.007	0.016	0.013	0.013	

***** Significant at $P < 0.05$ (AMOVA test); statistical probabilities derived from 10 100 permutations.

Supplementary Table 3 | Analysis of molecular variance (AMOVA test) based on a Φ_{ST} (genetic variance) matrix between haplotypes for the COI gene in the common periwinkle *L. littorea*. The samples were separated between northern (Norway), mid-range samples (UK and Roscoff) and southern samples (Île de Ré and Vigo) according to the identified climatic regions. Φ_{ST} = genetic variance within populations, Φ_{SC} = genetic variance among populations within groups, Φ_{CT} = genetic variance among groups.

Group	Source of variation	d.f.	SS	Variance components	Percentage of variation	Φ_{ST}	Φ_{SC}	Φ_{CT}
Climatic region	Among groups	2	19.657	0.118	13.98			0.140*
	Among populations within groups	4	2.941	0.003	0.04		0.000	
	Within populations	231	167.163	0.724	85.98	0.140**		
	Total	237	189.761	0.842				

* Significant at $P < 0.005$; ** Significant at $P < 0.0001$ (AMOVA test); statistical probabilities derived from 1023 permutations.

Supplementary Table 4 | Results of univariate ANOVAs testing for the effects of elevated P_{CO2} on the ion concentration in empty shells (n=30) of *L. littorea* across a latitudinal thermal gradient (46°-70°). Degrees of freedom (df), mean of square (MS), F-ratio (F) and probability level (p) are shown.

Trait	Source	df	MS	F	*P*
Calcium	**P_{CO2}**	**1**	**383231.580**	**88.821**	**< 0.0001**
	**Latitude**	**4**	**13150.407**	**3.048**	**0.019**
	**P_{CO2}*latitude**	**4**	**26931.384**	**6.242**	**< 0.0001**
Magnesium	**P_{CO2}**	**1**	**1.556**	**4.637**	**0.033**
	**Latitude**	**4**	**9.760**	**29.096**	**< 0.0001**
Strontium	**P_{CO2}**	**1**	**0.82**	**15.871**	**< 0.0001**
	**Latitude**	**4**	**0.103**	**19.989**	**< 0.0001**
	**P_{CO2}*latitude**	**4**	**0.23**	**4.384**	**0.002**
Potassium	**P_{CO2}**	**1**	**0.136**	**23.028**	**< 0.0001**
	**Latitude**	**4**	**0.102**	**17.303**	**< 0.0001**
Magnesium : Calcium	**P_{CO2}**	**1**	**1.389E-005**	**37.819**	**< 0.0001**
	**Latitude**	**4**	**1.506E-005**	**41.011**	**< 0.0001**
Magnesium : Strontium	P_{CO2}	1	0.091	0.366	0.546
	Latitude	4	12.709	51.032	**<0.0001**
Calcium : Strontium	**P_{CO2}**	**1**	**772074.445**	**679.717**	**< 0.0001**
	**Latitude**	**4**	**76561.623**	**67.403**	**< 0.0001**
	**P_{CO2}*latitude**	**4**	**3770.361**	**3.319**	**0.012**

Supplementary Table 5 | Mean mesocosm seawater physicochemical parameters. Mean (± SE) for seawater physico-chemical parameters measured or calculated during the duration of the experiment: oxygen concentration (O_2), salinity, temperature (°C), pH (NBS scale), dissolved inorganic carbon, total alkalinity (TA), carbon dioxide partial pressure (P_{CO2}), bicarbonate and carbonate ion concentration ($[HCO_3^-]$ and $[CO_3^{2-}]$), calcite and aragonite saturation state (Ω_{cal} and Ω_{ara}). * indicates parameters that were calculated using the CO2SYSs program[1] (, using dissociation constants[2,3] and $[KSO_4]$[4] . Different letters in the brackets indicate significant differences among treatments using Post Hoc Bonferroni test of one-way ANOVA ($p < 0.05$), according to 95% Confidence Interval test for Estimate Marginal Means (EMM) with Bonferroni correction[5].

Parameter	Ambient P_{CO2}	Elevated P_{CO2}
$[O_2]$ (mg L^{-1})	6.75 ± 0.01	6.68 ± 0.17
Salinity	34.77 ± 0.03	35.06 ± 0.04
Temperature (°C)	14.43 ± 0.06	14.89 ± 0.1
pH	8.03 ± 0.01[a]	7.67 ± 0.01[b]
TA (µequiv kg-1)	1596 ± 50	1596 ± 40
DIC (µmol kg-1)*	1726 ± 52	1627 ± 40
P_{CO2} (µatm)*	428 ± 17[a]	998 ± 30[b]
$[HCO_3^-]$ (µmol kg^{-1})*	1489 ± 47	1518 ± 38
$[CO_3^{2-}]$ (µmol kg^{-1})*	89.79 ± 3.5[a]	40.58 ± 1.3[b]
Ω_{cal}*	2.14 ± 0.08[a]	0.97 ± 0.03[b]
Ω_{ara}*	1.37 ± 0.05[a]	0.62 ± 0.02[b]

Supplementary References

1. Pierrot, D., Lewis, E. & Wallace, D. W. R. MS Excel program developed for CO2 system calculations, ORNL/CDIAC-105 (Carbon Dioxide Information Analysis Center, Oak Ridge National Laboratory, U.S. Department of Energy. Oak Ridge, TN, 2006).
2. Mehrbach, C., Culberson, C. H., Hawley, J. E., Pytkowicz, R. M. Measurement of the apparent dissociation constants of carbonic acid in seawater at atmospheric pressure. *Limnol. Oceanogr*. 18, 897-907 (1973).
3. Dickson, A. G. & Millero, F. J. A comparison of the equilibrium constants for the dissociation of carbonic acid in seawater media. *Deep-Sea Res*. 34, 1733-1743 (1987).
4. Dickson, A. G. Thermpodynamics of the dissociation of boric acid in synthetic seawater from 273.15 to 318.15 K. *Deep-Sea Res.* **37**, 755-766 (1990).
5. Melatunan, S. Biochemical, metabolic and morphological responses of the intertidal gastropod *Littorina littorea* to ocean acidification and increased temperature. PhD thesis, Plymouth University, UK (2012).
